# Editorial: Antimicrobial Usage in Companion and Food Animals: Methods, Surveys and Relationships With Antimicrobial Resistance in Animals and Humans, Volume II

**DOI:** 10.3389/fvets.2021.728267

**Published:** 2021-07-23

**Authors:** Lucie Collineau, Carolee A. Carson, Miguel A. Moreno

**Affiliations:** ^1^Epidemiology and Surveillance Unit, Laboratory of Lyon, French Agency for Food, Environmental and Occupational Health & Safety (ANSES), Lyon, France; ^2^Public Health Agency of Canada (PHAC), Guelph, ON, Canada; ^3^Department of Animal Health, Veterinary Faculty, Complutense University, Madrid, Spain; ^4^VISAVET Health Surveillance Centre, Complutense University, Madrid, Spain

**Keywords:** antibiotic use, metrics, livestock, pets, monitoring, surveillance

The best way to quantify antimicrobial use (AMU) in animals has raised wide research interests over the past years. Following the success of the first edition of the Research Topic on “Antimicrobial Usage in Companion and Food Animals: Methods, Surveys, and Relationships with Antimicrobial Resistance in Animals and Humans” Moreno et al., a second edition was launched. The objective was to continue the discussion on AMU metrics and expand the topic to other geographical regions (beyond North American and European Union countries), as well as other animal species (other than cattle, pigs, poultry, cats, or dogs).

A total of 14 articles contributed to this collection, including 12 original research papers and two review papers. Among the original research papers, geographical areas covered included Europe (*n* = 8), North America (*n* = 2), and Asia (*n* = 2). Animal species covered included pigs (*n* = 5), poultry (*n* = 4), multiple animal species (*n* = 3), dairy cattle (*n* = 1), dogs (*n* = 1), finfish aquaculture (*n* = 1), and horses (*n* = 1).

Out of the various research questions proposed in the Research Topic scope, the large majority of contributing studies aimed to compare different metrics to characterize AMU in animals (*n* = 10), while others primarily intended to compare AMU between countries (*n* = 4), between animal populations or farms, i.e., benchmarking (*n* = 3), or to monitor AMU trends over time (*n* = 1). None of the published studies addressed the aspects of linking AMU to antimicrobial resistance (AMR), or linking AMU between human and animal sectors, suggesting there is still room for more integrated and One Health approaches in the AMU metrics area.

Most studies relied on end-user (farms or veterinarians) data (*n* = 12), while only a few studies relied on national (*n* = 1) or supra-national data (*n* = 1). This suggests a recent shift from national to end-user data, which are closer to “actual” AMU. Interestingly, this shift was also described by Sanders et al. who reported the development of multiple farm-level monitoring systems in Europe and Canada over the recent years. These are public or private monitoring programs (~50% each), which for some of them manage to achieve full sector coverage.

Sanders et al. also reviewed the different AMU indicators being used by farm-level monitoring systems, defined as the amounts of antimicrobials consumed (numerator) normalized by the population at risk of being treated in a defined period of time (denominator). The authors demonstrated a clear lack of harmonization between farm-level indicators across countries and systems. The same observation was made by Narbonne et al. who systematically reviewed AMU indicators in finfish aquaculture. In addition, the calculation of AMU indicators in finfish aquaculture raised specific issues, e.g. related to the lack of average weight at treatment available in this sector.

Several contributing studies quantified the gap between different indicators applied to the same dataset, and discussed the impact this had on the study results. Depending on the indicator applied to broiler chicken and turkey farm-level data, Agunos et al. observed variations in reported quantity of use, temporal trends, and relative ranking of the antimicrobials. Discrepancies were also observed by Kuemmerlen et al. and O'Neill et al. when ranking Swiss and Irish pig farms using various AMU indicators, highlighting the fact that different methods of measuring AMU can affect a benchmarking system. Discrepancies appeared higher when comparing weight-based vs. dose-based metrics, while comparisons within dose-based metrics appeared relatively concordant. Similarly, Schnepf et al. reported little deviation when comparing Used Daily Doses with Defined Daily Doses in horses presented at a veterinary university clinic in Germany. Comparisons between populations, e.g., between countries, could be improved by applying a standardization procedure to correct for differences in the composition of livestock demographics, as suggested by Hommerich et al.

Some contributing studies also explored associations between AMU quantities and farm management practices. Caekebeke et al. studied associations between AMU and biosecurity levels in broilers and pig farms in Belgium and the Netherlands, and showed that Dutch farms overall had higher biosecurity and lower AMU than Belgian farms. In addition, Echtermann et al. reported positive significant associations between AMU and farm size, as well as between AMU in sows and piglets in Swiss farrow-to-finish pig farms. Olmos Antillón et al. explored variations in AMU between conventional and organic dairy farms in Sweden; while AMU for injectable and lactating cow intramammary treatments statistically differed between production systems, no difference was found for dry-cow therapies.

One study by Redding et al. looked at perceptions of AMU indicators by small and large animal veterinarians in the USA. While respondents were quite positive about being part of a benchmarking system, they also reported AMU indicators, and especially dose-based indicators to be confusing, and recommended further guidance on how to interpret the metrics. Hence, the authors stressed the importance of selecting AMU indicators that are meaningful to clinicians for AMU monitoring to have a positive impact on antimicrobial stewardship.

Beyond generating meaningful indicators, the issue of accessing detailed data that are necessary to calculate advanced indicators such as dose-based indicators was also raised. In their longitudinal study on AMU in Spanish dogs, Méndez and Moreno called for a pragmatic approach to use the simplest indicators based on the most frequently available information, as a compromise for permitting certain AMU data analyses. This is also the approach that has been used by Imam et al. and Barroga et al. in Bangladesh and the Philippines, where quantitative AMU data are not routinely recorded. The analysis of the proportion of farms using selected antimicrobial classes showed the frequent use of critically important antimicrobials in pigs and poultry in both countries, highlighting the critical need to improve antimicrobial stewardship in the region. Among others, this could be achieved via stronger AMU monitoring systems.

Between April 2019 and March 2021, there were substantial contributions (29 articles) to the two article collections. Both article collections highlighted the diversity of approaches to data collection and reporting of AMU information, with resulting implications for interpretation and communication of the findings. Within the article collections were themes of pragmatism in AMU reporting, a need for harmonization and transparency in documentation of methods, and reporting AMU in a way that is meaningful to the target audience to improve antimicrobial stewardship.

## Author Contributions

LC produced the first draft of the editorial. All authors edited and approved the editorial.

## Conflict of Interest

The authors declare that the research was conducted in the absence of any commercial or financial relationships that could be construed as a potential conflict of interest.

## Publisher's Note

All claims expressed in this article are solely those of the authors and do not necessarily represent those of their affiliated organizations, or those of the publisher, the editors and the reviewers. Any product that may be evaluated in this article, or claim that may be made by its manufacturer, is not guaranteed or endorsed by the publisher.

